# Professional Development for Teachers of Gifted Education in Hong Kong: Instrument Validation and Training Effectiveness

**DOI:** 10.3390/ijerph19159433

**Published:** 2022-08-01

**Authors:** Alan Chi Keung Cheung, Daniel Tan Lei Shek, Anna Na Na Hui, Kim Hung Leung, Ruby Shui Ha Cheung

**Affiliations:** 1 Centre for University and School Partnership, Faculty of Education, The Chinese University of Hong Kong, Hong Kong; wlkh22@yahoo.com.hk; 2 Department of Applied Social Sciences, The Hong Kong Polytechnic University, Hong Kong; daniel.shek@polyu.edu.hk; 3 Department of Social and Behavioural Sciences, City University of Hong Kong, Hong Kong; annahui@cityu.edu.hk; 4 Hong Kong Baptist University Affiliated School Wong Kam Fai Secondary and Primary School, Hong Kong; rshcheung@hkbuas.edu.hk

**Keywords:** professional development, school-based gifted education, Hong Kong, scale validation, program effectiveness

## Abstract

Project GIFT is a pioneer research-based gifted education program which has been found to be effective in fostering holistic development of students in Hong Kong. Nevertheless, little is known whether the Project is beneficial to teachers. To investigate the changes in teachers after participating in the Project, we adopted a quasi-experimental design with pretest and posttest data collected from experimental and control groups in this study. A total of 2031 primary and secondary school teachers participated in the professional development program of the Project. They completed validated measures on teachers’ knowledge of and attitudes toward gifted education, teaching behaviors, characteristics and competencies, in addition to well-being before and after participating in the program. Results of one-way ANCOVA showed that the program could promote teachers’ knowledge of gifted education and specific teaching strategies to gifted learners. This study provides preliminary support for the program in promoting holistic professional development of participating teachers in gifted education. The theoretical and practical implications of the findings are discussed.

## 1. Introduction

Gifted and talented students are often pushed aside in the educational system simply because most educators think that these students will perform well on their own without special or additional support. Nevertheless, these students need to be challenged and supported in order to reach their highest potential [1,2]. To provide adequate support to the gifted learners, professional training available for teachers to adopt specialized instructional practices such as differentiation and flexible grouping is a prerequisite [3]. Past research has demonstrated that teachers with professional training on gifted education is beneficial to gifted learners (e.g., [4,5,6,7]). Nonetheless, some studies have not revealed the association between teachers’ attitudes and behaviors, and the level of professional development of teachers [8,9]. As such, findings about the impact of professional development on teachers in gifted education are mixed and inconclusive. Furthermore, most of the existing studies examining the effectiveness of professional development programs aiming to support teachers in gifted education have been conducted predominately in the Western societies. To address these gaps, this study developed a professional development program of gifted education and examined its effectiveness among teachers in Hong Kong.

### 1.1. Professional Development for Teachers of Gifted Education

Nowadays, gifted education is prevalent across the globe because it contributes to fostering human capital and facilitating economic development in many societies [10]. Nevertheless, Reid and Horváthová [11] reviewed the current state of teacher education for the gifted in many European countries and unexpectedly found that teacher training on gifted education has been largely neglected, although great attention is paid to the education of the gifted. Besides, even though professional development programs are provided to teachers of gifted education in some countries, the programs have often targeted at only a portion of learning instructional objectives such as teachers’ pedagogy and self-confidence (e.g., [7,12]).

To fully promote the development of gifted students, teachers have to embrace changes in knowledge, attitude and instructional practices and to fully engage in professional development [13,14]. Although research studies on gifted education regarding characteristics and needs of gifted learners have been extensively conducted, teachers still hold myths about gifted students [15]. These myths discourage teachers to perform effective teaching strategies like acceleration for high ability learners, and subsequently prevent them from developing their potential [16]. For instance, Sheffield [17] revealed some myths about gifted mathematics students to teachers. The belief that ‘gifted mathematics students can develop on their own’ would restrict students’ understanding of Mathematics and hinder the development of their mathematical ability. Teachers’ mistaken beliefs in giftedness are likely to be developed as teachers do not possess sufficient knowledge and thorough understanding of giftedness [5]. As a result, the provision of research-based knowledge to teachers of gifted education via professional development programs is of paramount importance. In addition, changing teachers’ attitudes toward gifted education (as a prerequisite for improving pedagogy) and teaching skills for the gifted are also significant in planning professional development to the teachers of the gifted [18]. Consequently, a holistic professional development program targeted at improving teachers’ knowledge of and attitudes toward gifted education, and instructional strategies to the gifted is much needed. To our best knowledge, Edinger [19] made the only study which developed and implemented a holistic online program to enhance teachers’ knowledge, attitude, instructional practice, collaboration, and goal effectiveness in gifted education. Even though the findings have illustrated a positive change in each component for teachers six months after participating in the program, the validity and reliability of the measurement instrument have not been reported. Moreover, the questions regarding teachers’ professional knowledge, attitude and behavior were not systematically addressed. For example, the change in attitude was tapped by asking respondents about only the question of ‘To what extent did the online teacher professional development create positive change in your teaching attitude?’ Although a single-item measure could achieve the acceptable level of validity and reliability for a unidimensional construct, it is undesirable for researchers to use a single-item measure to assess teachers’ attitude toward gifted education and specific teaching practices, which could be better understood in terms of multiple dimensions [15,20]. In sum, a holistic professional development program for teachers of gifted learners accompanied with validated evaluative tools has been lacking. To address these gaps, this study aimed at developing a holistic professional development program to teachers of regular class and gifted students and creating psychometrically sound measurement instruments for program evaluation in Project GIFT.

### 1.2. Project GIFT

In 2016, the Project ‘Jockey Club “Giftedness into Flourishing Talents” Project’ (Project GIFT) was launched to promote school-based gifted education in Hong Kong. It was solely funded by the Hong Kong Jockey Club Charities Trust. Apart from developing enriched and differentiated curricula for students, the Project provided professional training in school-based gifted education to teachers. There were 20 project schools (experimental schools) and 8 non-project schools (control schools) joining the Project. Further details of the Project can be seen in [21].

#### 1.2.1. Development and Implementation of the Professional Development Program

In Project GIFT, the professional development program for gifted education teachers was aimed at enhancing teachers’ knowledge of gifted education and characteristics of gifted students, fostering positive attitudes toward school-based gifted education, and promoting the use of specific instructional practices like differentiation.

There are several conceptual pillars for the teacher development of the program. First, Chan’s [22] University-School Tripartite Model of Talent Development suggests universities could be the centers for training teachers and the development of curriculum materials to the gifted. With close collaboration between university and school, teacher training workshops targeted at preparing teachers to develop gifted programs and activities in their home schools, and to adopt the ‘gifted and talented’ approach in their teaching are provided to teachers by universities. As such, teachers’ knowledge of gifted education and characteristics of gifted students, and the use of specific instructional practices like differentiation and flexible grouping could be promoted. Second, based on the experience of the university-school partnership, Lee [23] developed a 4-P model with the use of action research and reflective practice, which aimed to promote reflection and professional competency of teachers. The model consists of four main processes (problem clarification, planning, progress action, and progress evaluation) which constitute a cycle. At first, teachers discover and understand the problems of their teaching. Then they refine their problems by using applied research and implement the revised curriculum that is collaboratively developed by teachers and university staffs. In the process, professional support and advice are provided to teachers in collaborative lesson preparation, lesson observation, and post-lesson discussion. Finally, teachers reflect on their teaching and adopt further refinement if necessary. Under the 4-P model, teachers would improve their instructional practices to the gifted continuously and develop positive attitudes toward gifted education through their successful teaching experiences. Moreover, Lee advocated to develop a professional learning community between universities and schools, and among schools. As such, teachers can share their successful teaching experiences with each other and develop positive attitudes toward school-based gifted education.

Third, based on the schoolwide enrichment model (SEM, [24]), organizational components including the development of a schoolwide enrichment team and network, training schoolwide enrichment teaching specialists, and adoption of a democratic school management plan in gifted education are established in project schools.

With reference to the University-School Tripartite Model of Talent Development, the 4-P Model and the organizational components of the SEM, Project GIFT utilized resources of the university and provided different types of support to teachers in the experimental schools. The support could be divided into two categories including specific school support initiatives and general teaching development program. They would help teachers implement enriched and differentiated curricula, which were developed based on Renzilli’s three-ring conception of giftedness [24], in their schools effectively. According to three-ring conception of giftedness, above average ability, high level of task commitment and creativity are three main elements for creating gifted behaviors of students. As such, training activities such as creativity and affective education workshops and STEM seminars were organized to teachers. These activities could assist teachers in designing enriched and differentiated curricula to promote students’ creativity, higher-order thinking skills and motivation.

##### Specific School Support Initiatives

School Development Officers of Project GIFT ‘walked with’ and accompanied teachers throughout the project period from the start-up to the completion. Considering organizational components of the SEM, the project staff assisted schools in setting up Gifted Education task forces and understanding the talent and characteristics of students by using different criteria and instruments. After identifying the strengths and interests of students and preparing a database on school information, intensive onsite school visits were then provided to schools for planning and implementing the program based on the findings. Aligned with the 4-P model, the project staff helped teachers to discover and understand any issues in the original curriculum and helped modify the design of school-based gifted education programs for students collaboratively in lesson planning sessions. During lesson observation and post-lesson discussion, School Development Officers and Advisors provided professional advice to teachers for improving the lessons.

##### General Teaching Development Program

Echoing the University-School Tripartite Model of Talent Development, thematic seminars and workshops, lectures, overseas study trips, academic conference experience, and cluster professional sharing activity were organized and provided to teachers of the experimental schools by the University. The details of the activities are illustrated in Table 1.

The provision of specific school support initiatives and general teaching development program was consistent with the basic notion of the Dynamic Scaffolding Model of teacher development in gifted education [25], which highlights ongoing support and training workshops are essential to help teachers gain knowledge about giftedness and guide teachers to discover optimal way to design and implement gifted education programs to their gifted learners.

#### 1.2.2. Evaluation of the Program

Based on Clarke and Hollingsworth’s [26] interconnected model of professional growth, changes in personal (knowledge, belief, attitude) and practice domains of teachers during professional learning are examined. As the instruments for assessing the change in teachers’ knowledge, attitude and behaviors after professional development for teachers in gifted education are scarce, we utilized the Knowledge-Attitudes-Behaviors (KAB) framework [27] to develop the measures to assess teachers’ knowledge, attitudes, and behaviors in gifted education. It is because successful outcomes of professional development in education involve knowledge gains and the development of positive attitudes and behaviors.

A two-group pretest-posttest design was utilized to examine the impact of the professional development program on teachers. There were twenty Hong Kong primary and secondary schools in the experimental groups (N = 1510). For the control groups, there were eight Hong Kong primary and secondary schools (N = 521) which had similar characteristics as the schools in the experimental groups. In this study, we hypothesized that teachers in the experimental groups would have better outcomes at posttest than those of the control group teachers when controlling for the pretest scores (i.e., ANCOVA).

## 2. Materials and Methods

### 2.1. Participants

A total of 2031 Hong Kong in-service teachers were recruited in this study. They came from 28 schools which were selected according to purposive sampling. Schools showing strong commitment to gifted education, admitting students with diverse academic abilities, having a significant number of disadvantaged students, and being supported by different sponsoring bodies were selected as experimental schools. Schools which possessed similar characteristics of the experimental schools (e.g., school banding, medium of instruction, and religious background) and teachers (e.g., years of teaching experience) were chosen as control schools. At last, the experimental groups consisted of 15 primary schools and 5 secondary schools while the control groups included 6 primary schools and 2 secondary schools. As teachers did not write down their names in the questionnaires and no concrete identifiers (only the day and the month of their birthday and the surnames of their mothers were used as the identifiers) were utilized, the questionnaire at pretest did not successfully match with those at the posttest for some teachers. Finally, there were 734 teachers in the matched sample. The effectiveness of the professional development program on teachers was examined based on the data of the matched sample. The demographic information of teachers in the experimental and control groups of the matched sample is illustrated in Table 2.

### 2.2. Instruments

In this study, the measure on teachers’ knowledge of and attitudes toward gifted education, and specific teaching behaviors to the gifted were newly developed and utilized. Basically, a pool of items sufficient for the measure was initially created based on relevant instruments used in previous literature. Afterwards, items were selected from the item pool for the preliminary version of the measure using expert panel reviews who have extensive research and teaching experiences (more than 30 years) in the field of gifted education. Some items were deleted based on a prior criteria, such as too lengthy, lack of clarity and conciseness, questionable relevance, or undesirable similarity to other items [28]. After incorporating the comments provided by the experts, the most representative items were retained. Subsequently, the psychometric properties of the measure were examined. Apart from these measures, teachers’ characteristics and competencies to nurture gifted learners, and teachers’ well-being were also explored. In addition, demographic information like age, gender, grade, subject taught, years of teaching experience, highest education level attained, professional training, and experience in teaching gifted learners were recorded. The measures are briefly described as follows.

#### 2.2.1. Teachers’ Knowledge of Gifted Education

A 10-item Teacher Knowledge Scale (TKS) was developed based on the common myths about gifted children [29] and the policies of school-based gifted education in Hong Kong [30]. Examples of items include “Only students who have high intelligence (IQ score over 130) are classified as gifted students” and “The Education Bureau considers gifted education should be provided to every student who has outstanding performance in different areas”. Respondents were asked to assess the statements by choosing one of three response options: true, false, and not sure. Among the 10 statements, four are true whereas six are false. One mark was assigned to the right answer. No mark was given to the incorrect answer and those marked as undecided (see [31]). Higher total score means a higher level of knowledge of the characteristics of gifted education and gifted students.

#### 2.2.2. Teachers’ Attitudes toward School-Based Gifted Education

A 12-item Teacher Attitude Scale (TAS) was constructed based on the instruments for assessing teachers’ opinions on gifted education in past studies [32,33,34,35,36]. It is composed of three dimensions including teacher support (5 items, e.g., The Education Bureau provides adequate training in gifted education for teachers), opposition to gifted education (4 items, e.g., Implementing gifted education will hugely increase teachers’ workloads), and support for gifted students (3 items, e.g., I should cater for the special educational needs of gifted students). Participants were asked to respond the items on a 5-point scale (1 = strongly disagree; 5 = strongly agree). The composite reliability of the subscales of teacher support, opposition to gifted education, and support for gifted students were 0.84, 0.68 and 0.57, at pretest, and 0.84, 0.73, and 0.68 at posttest, respectively.

#### 2.2.3. Teachers’ Behavior to the Gifted Learners

A 12-item Teacher Behavior Scale (TBS) was developed based on the instruments for assessing instructional practices to the gifted in previous studies [37,38,39]. It consists of three dimensions including nurturance for gifted students (5 items, e.g., I search for suitable resources for gifted students who need support), differentiated teaching (4 items, e.g., I respond to the learning needs of high-ability or gifted students with tiered assignments.), and learning support for regular students (3 items, e.g., I cultivate students’ higher-order thinking with high-level questions). Respondents were asked to answer the items on a 5-point scale (1 = least like me; 5 = most like me). The composite reliability of the subscales of nurturance for gifted students, differentiated teaching, and learning support for regular students were 0.84, 0.71 and 0.75 at pretest, and 0.85, 0.78, and 0.79 at posttest, respectively.

#### 2.2.4. Teachers’ Well-Being

A 29-item Teacher Well-Being Scale (TWBS) was used to assess the psychological and subjective well-being of teachers. Items of the TWBS were derived from the 6-factor Psychological Well-Being Scale (PWBS, [40]) and the unidimensional Satisfaction with Life Scale (SWLS, [41]) (Appendix A). Respondents were asked to indicate the extent to which they agreed to each statement along a 5-point scale with response options ranging from 1 (= strongly disagree) to 5 (= strongly agree). Previous research with Chinese samples has shown that the PWBS and the SWLS are valid and reliable (e.g., [40,41,42]. The composite reliability of the seven subscales ranged from 0.83 to 0.92 in this study.

#### 2.2.5. Characteristics and Competencies of Teachers of Gifted Students

A 25-item Teacher Characteristics and Competencies Scale (TCCS) was developed by David Chan and Lai Kwan Chan (Appendix B), who contributed to initial development and implementation of the project in this study. It was utilized to examine important attributes and skills such as professional predispositions and teaching competencies for teachers of gifted students. Respondents were required to answer on a 5-point scale (1 = do not possess; 5 = totally possess). The composite reliability of the five subscales ranged from 0.73 to 0.86 in this study.

### 2.3. Procedures

In this study, we compared the outcome variables in the experimental groups with the control groups. At first, we sent an invitation letter accompanied with the project outline to all primary and secondary schools in Hong Kong. Afterwards, twenty schools among seventy respondents were purposively chosen as the experimental schools in August 2017. Written consent was obtained from the teachers who were willing to take part in the Project. Teachers of the experimental groups completed the assessment at two different times between November 2017 and July 2019. At pretest, each teacher completed a questionnaire containing five scales and demographic variables. He/She was required to write down the date and the month of his/her birthday, and the surname of his/her mother as identifiers to match the pretest with the posttest results. The questionnaires administered at posttest was the same as those administered at pretest. We purposively selected eight control schools in January 2019. Control teachers completed the same assessment as did the experimental teachers between February 2019 and June 2019. The assessment method and the time lag between two assessments were identical as those for the experimental teachers. The attrition rate of the whole sample was 1.5%.

### 2.4. Data Analysis

As the average missing rate per item was 0.23%, no imputation was performed. Data analysis was conducted with listwise deletion. Regarding the scale development and validation for the TAS and the TBS, exploratory factor analysis (EFA) and confirmatory factor analysis (CFA) were carried out to explore and validate the factor structure of the TAS and the TBS. At the same time, CFA was conducted to investigate the psychometric properties of the TWBS and the TCCS. The overall model fit was evaluated based on non-normed fit index (NNFI): ≥0.95 = satisfactory fit and comparative fit index (CFI): ≥0.95 = satisfactory fit [43], root-mean-square error of approximation (RMSEA): <0.05 = a close fit, 0.05–0.08 = a fair fit, 0.08–0.10 = a mediocre fit and >0.10 = a poor fit [44], and standardized root-mean-square residual (SRMR): <0.08 = a good fit [45]. To assess the impact of program intervention on each of the outcome variable of the sample, one-way ANCOVA was utilized to compare posttest scores between the experimental groups and the control groups after controlling for pretest scores. The present Chi-square test results revealed that the experimental and control groups did not differ in the proportion of age group (*p* = 0.396), gender (*p* = 0.869), years of teaching experience (*p* = 1.00), education level (*p* = 0.773), professional training (*p* = 1.00), and years of teaching experience to gifted students (*p* = 0.777). Bonferroni correction method was not utilized to adjust *p*-values in multiple-testing process in the present study as we treated and analyzed outcome variables individually (see [46]). Therefore, a *p*-value less than 0.05 implies a significant difference between two means at posttest. Cohen’d was used to assess the effect size (0.2 as small, 0.5 as medium, and 0.8 as large [47]). EFA and ANCOVA were conducted using SAS 9.4 and SPSS 26.0, respectively. CFA was performed using Lisrel 8.54 with maximum likelihood estimation.

## 3. Results

### 3.1. Scale Validation

#### 3.1.1. Teacher Knowledge Scale (TKS)

Content validity of the TKS was well-supported by expert reviews panel that was composed of academics who have been working in gifted education for many years (>30 years). The experts assured good representativeness and wide coverage of the items related to the misconceptions about gifted education and gifted students in the TKS (Table 3). Besides, the present results revealed that Kuder-Richardson 20 of TKS was 0.44 and 0.52 at pretest and at posttest, respectively. As such, the reliability of the TKS was marginally adequate and moderate (see [48,49]).

#### 3.1.2. Teacher Attitude Scale (TAS)

Regarding the TAS, all items except one were normally distributed because the absolute values of univariate skewness (ranged from 0.05 to 1.28) and kurtosis (ranged from 0.07 to 1.11) values were less than 2 [50]. The non-normal item was removed and 17 items were proceeded to EFA. As the value of Kaiser-Mayer-Olkin measure of sampling adequacy was 0.79 (higher than 0.50 cutoff value) and the value of Bartlett’s test of sphericity was 3019.7 with 136 degree of freedom (*p* < 0.05), the data was adequate for performing factor analysis [51]. EFA utilizing principal axis factoring was carried out to explore the factor structure of the TAS. The results indicated a three-factor structure of the TAS. The factors had eigenvalues of 3.30, 2.05, and 1.42, which were greater than 1.0 cutoff value. Among the 17 items, 5 items were subsequently removed because of small factor loading or being the constitutes of the meaningless factor. EFA was re-run and all the 12 items ended up loading on three factors without cross-loading [52]. Inspection of factor loadings revealed that all loadings ranged from 0.36 to 0.88. The three-factor model explained 56.34% of the total scale variance. The present CFA results further confirmed the three-factor structure of the TAS (χ2 = 170.95, df = 51, *p* < 0.001, NNFI = 0.95, CFI = 0.96, RMSEA = 0.058, SRMR = 0.051) (also see Table 4).

#### 3.1.3. Teacher Behavior Scale (TBS)

In regards to the TBS, all items were normally distributed because the absolute values of univariate skewness (ranged from 0.01 to 0.66) and kurtosis (ranged from 0.01 to 0.62) values were less than 2. All the 13 items were proceeded to EFA. As the value of Kaiser-Mayer-Olkin measure of sampling adequacy was 0.91 and the value of Bartlett’s test of sphericity was 3515.9 with 78 degree of freedom (*p* < 0.05), the data was adequate for conducting factor analysis. EFA utilizing principal axis factoring was carried out to derive the factor structure of the TBS. The results showed a three-factor structure of the TBS. The factors had eigenvalues of 5.01, 1.33, and 1.05, which were greater than 1.0 cutoff value. Among the 13 items, one item was deleted because of very low factor loading. EFA was re-run and all the 12 items ended up loading on three factors without cross-loading. Inspection of factor loadings of the items revealed that all loadings ranged from 0.47 to 0.69. The three-factor model explained 61.56% of the total scale variance. The present CFA results validated the three-factor structure of TBS (χ2 = 341.17, df = 51, *p* < 0.001, NNFI = 0.94, CFI = 0.96, RMSEA = 0.094, SRMR = 0.056) (also see Table 5).

In sum, the TKS, the TAS and the TBS are valid and reliable instruments. They were subsequently used to assess the impact of the professional development program of Project GIFT on teachers. Moreover, the present CFA results supported the factorial validity of 7-factor model of TWBS (χ2 = 1433.01, df = 356, *p* < 0.001, NNFI = 0.97, CFI = 0.98, RMSEA = 0.068, SRMR = 0.048), and 5-factor model of TCCS (χ2 = 1617.68, df = 265, *p* < 0.001, NNFI = 0.95, CFI = 0.96, RMSEA = 0.090, SRMR = 0.064).

### 3.2. Effectiveness of Professional Development to Teachers

Descriptive statistics and Cronbach’s alphas for all outcome variables from the experimental and control groups are illustrated in Table 6. The reliability of each scale was acceptable (Cronbach’s alphas ranged from 0.56 to 0.96) (see [53,54]).

**Figure 1:**
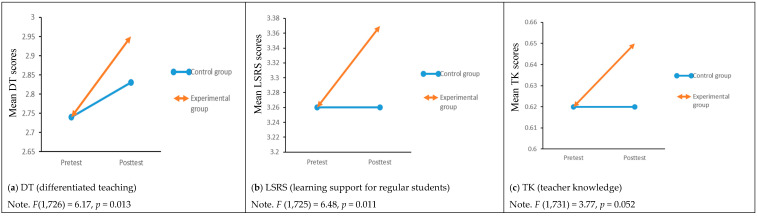
Graphs showing the difference in outcome variables at posttest from one-way ANCOVA between experimental and control groups.

The results of one-way ANCOVA revealed significant difference(s) in posttest scores on differentiated teaching, *F*(1,726) = 6.17, *p* < 0.05; learning support for regular students, *F*(1,725) = 6.48, *p* < 0.05, and teacher knowledge about gifted education, *F*(1,731) = 3.77, *p* = 0.05 (marginally significant). The difference(s) in each outcome variable is graphically presented in Figure 1a–c.

The results of the post hoc analysis in Table 7 indicated that the experimental groups has greater adjusted mean scores in differentiated teaching (d = 0.24), learning support for regular students (d = 0.22), and teacher knowledge about gifted education (d = 0.16) than those of the control groups.

## 4. Discussion

Professional training for teachers in gifted education is a prerequisite for the provision of high-quality curriculum and instruction for gifted students [55]. Nevertheless, teacher training for gifted education has been largely ignored in many countries [56]. Therefore, the present study developed and implemented a holistic professional development program for teachers of gifted education in Hong Kong. One of the major strengths of this study was that it conducted holistic investigation of cognitive (knowledge), affective (attitudes), and behavioral effects of professional development on teachers. This outperforms previous gifted education studies on teacher training, which have focused on the change in only a portion of instructional learning objectives to teachers (e.g., [7,57]). Second, this study utilized the experimental design with the control groups to examine the impact of professional development on teachers. This enhances the methodological rigor of past gifted studies which were commonly descriptive and correlational in nature [58]. Third, a large sample was utilized to enhance the credibility of the findings in the present study [59]. Fourth, in view of limited research on the professional development for teachers of gifted education in the Chinese context, our study adds on the literature of teacher training on gifted education in Asian countries.

In this study, the findings were generally positive and they were in line with most of the original expectations. Since two scales and one objective test were developed based on three instructional learning outcomes of the KAB framework, the validity and reliability analysis of these measures offered us significant feedback on the alignment of the outcomes of knowledge, attitude and behavior with teacher professional development in gifted education of the Project. The findings of this study provided empirical support not only for the psychometric properties of the TKS, the TAS and the TBS, but also for the multidimensionality of the constructs: teacher attitude toward school-based gifted education and specific instructional practices to the gifted. To our best knowledge, research on scale development and validation for the assessment of teachers’ knowledge, attitudes and behaviors in gifted education is limited and unsatisfactory. For instance, psychometric properties of the scale for assessing teacher attitude about gifted education were unreported in [60] or were undesirable because of low alpha and loading values in [15], which renders the authors to admit that the instrument in the present form could assist in program planning rather than as a tool for statistical analysis and program evaluation. This study advances past gifted studies on scale development and validation by creating psychometrically sound TAS and TBS.

Based on the self-report data by the teachers in the survey, it could be summarized that teachers’ knowledge of gifted education and gifted students and specific teaching strategies to the gifted were generally improved after participating in Project GIFT. The results were consistent with [19] which revealed positive changes in content knowledge and teaching practices of teachers after participating in the professional development program of gifted education. As stated by [61], differentiated teaching practices are underutilized by teachers of the gifted and regular classroom teachers, especially in middle school classrooms. The present professional development program could offer a promising training to enhance the use of differentiation in the classroom for primary and secondary school teachers in Hong Kong. Moreover, as teaching practices for gifted learners and regular classroom students constitute two distinct factors in the TBS, it implies that teachers acknowledge the necessity of providing additional, specialized instructional strategies to assist the gifted in developing their potential. To assist teachers in providing various learning opportunities to the gifted, more evidence-based programs which have been proved as effective are needed. It is because teachers’ perception of the effectiveness of teacher training in gifted education is crucial to their implementation of gifted education in regular classrooms and their perceived competency to teach gifted students [62]. The positive outcomes of the present program undoubtedly supported the fact that the program might serve as one of the useful trainings for teachers of gifted learners in Hong Kong.

Nonetheless, there was no improvement in teachers’ attitudes toward school-based gifted education. As stated by [8], the impact of professional training on teachers’ attitudes toward gifted education is still inconclusive. Training or experience in gifted education is not indicative of more favorable attitudes toward the gifted. It may because there are other crucial factors influencing teachers’ attitudes toward gifted education such as school support, principal leadership, and adequacy of resources available for gifted education. Future research should take the intervening effects of different contextual factors in schools into account when assessing the impact of professional development on teachers’ attitudes toward school-based gifted education.

Besides, there was no change in teachers’ well-being and teachers’ predispositions. It might be attributed to the fact that the promotion of teachers’ well-being and predispositions were not the main foci of the professional development program of the Project. Teacher well-being may also be influenced by other factors such as work stress and work environment. Future research should incorporate affective and personality elements such as teaching efficacy in gifted education and creativity teaching into the training program, and subsequently evaluate their impact via developing content-valid measures (see [9]).

## 5. Implications

Several theoretical and practical implications of the findings are stated as below. First, research on the 4-P model has mainly focused on its benefits to students (e.g., [63]) and its impact on teachers has rarely been explored. The findings of this study empirically supported the benefits of the 4-P model to promote teachers’ knowledge of gifted education and specific instructional strategies to gifted learners. Second, even though the application of SEM in gifted education has been found to be associated with positive student and teacher outcomes (e.g., [64,65]), there is a paucity of research to indicate its efficacy in studies using a large sample [66]. We recruited a large number of teachers to participate in the study. This enhances the power of the positive findings of this study and further reaffirms the feasibility and value of SEM in gifted education.

Practically, in keeping with the standards by [67], which state that gifted education specialists should actively take part in professional training to increase their professional knowledge and enhance their teaching effectiveness, this study offers an evidence-based professional development program to teachers in gifted education. In response to the findings by [68,69] which revealed that the myths and incorrect beliefs about giftedness still existed and could be eliminated via teacher training, the evidence-based professional development program of this study could promote the knowledge of teachers in gifted education. This evidence-based training program could be used by Hong Kong teachers in the following ways. The program could be recommended to schools as professional development activities in their Teacher Development Days. Also, the program could be recommended to the Education Bureau as the in-service training program for teachers and school principals who are interested in school-based gifted education. This training program could enhance the confidence and the capability of teachers to implement gifted education in their schools. Moreover, the contents of the training program could be added into undergraduate teacher education. This could enhance student teachers’ knowledge of gifted education and help student teachers develop high efficacy beliefs regarding the implementation of school-based gifted education. Apart from local contribution, the program might serve as the implementation framework for other Asian countries in advancing teachers’ professionalism in and capacity for gifted education.

Besides, the present study developed psychometrically sound instruments that probes teachers’ knowledge, attitudes, and behaviors in gifted education. It could be adopted to assess the effectiveness of teacher training in a holistic manner for pre-service and in-service teachers. The instruments also allow the researchers to focus on multiple dimensions that contribute to teachers’ attitude and behaviors, which would l be useful for designing specialized professional development programs and determining program goals. Moreover, as the present findings revealed that the instruments showed good psychometric properties, it could be used in other Asian contexts and acts as objective reference instruments for related research in the international context.

## 6. Limitations

There are several limitations to be addressed. First, as teachers were not randomly assigned to the experimental and control groups, the present results should be viewed as preliminary. Even though the results of this study revealed the beneficial impact of the professional development program on teachers, future research should utilize randomized controlled trials to replicate this study. Second, although the sample size of this study can be seen as respectable, it is desirable to recruit more teachers with different background characteristics. It is because the participants of this study were restricted to primary and secondary in-service teachers in Hong Kong. For instance, [9,12,70] have called for the provision of professional development in gifted education to pre-service teachers because university level trainings in gifted education available for pre-service teachers are very limited. Third, as the present findings were limited to Hong Kong teachers, we would not generalize the present results to teachers in other Chinese communities. Future research should replicate this study in other Chinese contexts. This would enhance the credibility of the findings of this study. Fourth, the present findings only revealed the short-term effects of the professional development program because the assessment was conducted at two time points only (i.e., pretest and posttest). Future research should adopt a longitudinal design and collect more data at different time points. As such, researchers are able to know the long-term effects of teacher professional development (see [71]).

## 7. Conclusions

This study responded to several limitations in the existing literature on the development and evaluation of professional development in gifted education to teachers. Conceptually, we integrated different models like the 4-P model and the schoolwide enrichment model to develop a pioneer professional development program to teachers in gifted education. Methodologically, we utilized newly-developed instruments with good psychometric properties in a quasi-experimental study. Consistent with our expectations, this study revealed that teachers in the experimental groups showed positive changes after participating in the professional development program. As such, the benefits of Project GIFT in promoting professional competency of teachers in gifted education are shown.

## Figures and Tables

**Table 1:** Teacher training program of Project GIFT.

Type of the Activity	Number of Sessions	Content of the Activity
1. Thematic seminars and workshops	8	A series of academic lectures under the theme of “Talent development—Nurturing creativity and excellence”, and workshops on differentiation and action research
2. Centralized professional development workshops	26	Staff development on creativity and creative teaching, as well as affective education to gifted and regular students
3. Overseas study trips	2	Study trips to Taiwan and Singapore
4. Academic conferences	2	Participation in The 15th Asia Pacific Conference on Giftedness and WCGTC World Conference
5. Cluster professional sharing	58	Topics such as the implementation of school-based gifted education and pull-out programs, gifted education in English Language Education and STEM education, and utilization of e-learning in gifted education

**Table 2:** Demographic information of the participants.

	Matched Sample (N = 734)	
	Experimental (N = 548)	Control (N = 186)
Gender		
Male	129 (23.5%)	47 (25.3%)
Female	419 (76.5%)	139 (74.7%)
Age (median)	39.50	40.98
Years of teaching experience (median)	15.93	16.18
Grade		
Primary	387 (70.6%)	139 (74.7%)
Secondary	161 (29.4%)	47 (25.3%)
Education level		
Diploma and Associate degree	15 (2.7%)	5 (2.7%)
Bachelor	306 (55.8%)	109 (58.6%)
Master and Doctorate	227 (41.4%)	72 (38.7%)
Professional training		
Yes	524 (95.6%)	179 (96.2%)
No	23 (4.2%)	7 (3.8%)
Experience in teaching gifted students		
Yes	263 (48.0%)	87 (46.8%)
No	285 (52.0%)	99 (53.2%)

Note. One respondent did not indicate his professional training in experimental groups.

**Table 3:** Items of the Teacher Knowledge Scale.

1. The Education Bureau considers gifted education should be provided to every student who has outstanding performance in different areas.
* 2. The Education Bureau implements a 3-tier operational mode in gifted education with the level one as off-site support.
* 3. Only students who have high intelligence (IQ score over 130) are classified as gifted students.
4. Gifted education should be part of quality education, with the aim of nurturing the potential and talents of every student.
* 5. The serving target of gifted education is only limited to high-ability or gifted students.
6. The academic performance of gifted students and their actual ability may not be consistent.
* 7. Every student is gifted, but with different areas of giftedness.
8. Some gifted students are with Dyslexia.
* 9. Through hard work, all students can become gifted individuals.
* 10. A twice-exceptional gifted student refers to a student who has two types of gifted features.

Note. Chinese items are paraphrased in English. * false items.

**Table 4:** Factor loadings of the12 items of the Teacher Attitude Scale at pretest of the matched sample.

Dimensions and Items	Factor Loadings
Teacher Support	
1. In general, schools in Hong Kong effectively implement gifted education.	0.55
2. The Education Bureau provides sufficient resources for schools to implement gifted education.	0.75
3. The Education Bureau provides schools with necessary professional support to implement gifted education.	0.89
4. The Education Bureau provides adequate training in gifted education for teachers.	0.77
5. Our school provides adequate support for teachers responsible for nurturing gifted students.	0.58
Opposition to gifted education	
6. Implementing gifted education will hugely increase teachers’ workloads.	0.76
7. Implementing gifted education will cause difficulties to everyday teaching.	0.81
8. Taking care of gifted students in class will negatively affect other students’ learning.	0.37
9. With huge learning diversity among students, teachers cannot fulfil the educational needs of gifted students in everyday teaching.	0.36
Support for gifted students	
10. I should have a greater understanding of the characteristics and needs of gifted students.	0.57
11. It is the responsibility of each teacher to provide counseling and support to gifted students who have behavioral or emotional problems.	0.53
12. I should cater for the special educational needs of gifted students.	0.57

Note. Chinese items are paraphrased in English. Factor loadings are standardized estimates and significant at 0.05 level.

**Table 5:** Factor loadings of the12 items of the Teacher Behavior Scale at pretest of the matched sample.

Dimensions and Items	Factor Loadings
Nurturance for gifted students	
1. I select appropriate teaching materials for high-ability or gifted students.	0.73
2. I search for suitable resources for gifted students who need support.	0.76
3. I provide training or counseling activities for high-ability and gifted students.	0.74
4. Through acceleration, high-ability or gifted students in my class can learn at a different pace.	0.64
5. I design pull-out programs or activities for high-ability students to have a deeper understanding of certain topics.	0.68
Differentiated teaching	
6. I use differentiated teaching through appropriate grouping of students based on their abilities or traits.	0.47
7. I use curriculum compacting for high-ability students so that they have time for self-learning and project-based learning.	0.72
8. I provide opportunities for students to choose learning activities related to the core curriculum according to their interests.	0.72
9. I respond to the learning needs of high-ability or gifted students with tiered assignments.	0.55
Learning support for regular students	
10. I immerse three core elements advocated in gifted education (higher-order thinking skills, creativity and personal-social competence) in my everyday teaching.	0.66
11. I cultivate students’ higher-order thinking with high-level questions.	0.74
12. I arrange enquiry-based learning activities to nurture students’ creativity and higher-order thinking ability.	0.72

Note. Chinese items are paraphrased in English. Factor loadings are standardized estimates and significant at 0.05 level.

**Table 6:** Descriptive statistics and reliabilities of all outcome variables for teachers in the experimental and control groups.

	Experimental Groups				Control Groups			
Variables	Pretest		Posttest		Pretest		Posttest	
	M(SD)	α	M(SD)	α	M(SD)	α	M(SD)	α
Teacher knowledge	0.62 (0.17)	-	0.65 (0.18)	-	0.63 (0.16)	-	0.62 (0.17)	-
Teacher attitudes								
TS	2.69 (0.66)	0.83	2.84 (0.70)	0.85	2.66 (0.69)	0.85	2.77 (0.63)	0.83
OGE	3.23 (0.62)	0.65	3.18 (0.67)	0.72	3.31 (0.62)	0.65	3.20 (0.63)	0.69
SGS	3.75 (0.62)	0.56	3.72 (0.67)	0.69	3.79 (0.60)	0.61	3.74 (0.56)	0.61
Teacher behaviors								
NGS	2.71 (0.62)	0.82	2.84 (0.69)	0.85	2.65 (0.66)	0.85	2.74 (0.58)	0.83
DT	2.75 (0.60)	0.70	2.94 (0.68)	0.79	2.73 (0.63)	0.72	2.82 (0.60)	0.74
LSRS	3.25 (0.60)	0.75	3.35 (0.66)	0.81	3.29 (0.56)	0.71	3.28 (0.55)	0.70
Teacher well-being	3.64 (0.50)	0.95	3.66 (0.54)	0.96	3.63 (0.49)	0.94	3.61 (0.46)	0.95
Teacher characteristics	3.54 (0.44)	0.90	3.57 (0.49)	0.92	3.56 (0.47)	0.91	3.58 (0.43)	0.91
Teacher competency	3.16 (0.53)	0.92	3.30 (0.57)	0.93	3.19 (0.54)	0.92	3.27 (0.56)	0.93

Note. TS = teacher support, OGE = opposition to gifted education, SGS = support for gifted students, NGS = nurturance for gifted students, DT = differentiated teaching, LSRS = learning support for regular students.

**Table 7:** Results of one-way ANCOVA on the outcome variables for teachers in the experimental and control groups.

			Experimental Groups	Control Groups			
Variables	N for the Analysis	Pretest	Posttest	Posttest			
		M	M(SE)	M(SE)	*F*-Value	df	*p*-Value
Teacher knowledge	734	0.62	0.65 (0.01)	0.62 (0.01)	3.77	1, 731	0.052
Teacher attitudes							
TS	732	2.68	2.83 (0.03)	2.78 (0.04)	1.04	1, 729	0.309
OGE	733	3.25	3.19 (0.02)	3.16 (0.04)	0.40	1, 730	0.526
SGS	733	3.76	3.73 (0.02)	3.73 (0.04)	0.00	1, 730	0.997
Teacher behaviors							
NGS	729	2.70	2.84 (0.02)	2.77 (0.04)	2.65	1, 726	0.104
DT	729	2.74	2.95 (0.02)	2.83 (0.04)	6.17	1, 726	0.013
LSRS	728	3.26	3.37 (0.02)	3.26 (0.04)	6.48	1, 725	0.011
Teacher well-being	733	3.64	3.66 (0.02)	3.62 (0.03)	1.39	1, 730	0.238
Teacher characteristics	733	3.54	3.58 (0.02)	3.57 (0.03)	0.06	1, 730	0.812
Teacher competency	731	3.17	3.31 (0.02)	3.26 (0.04)	1.73	1, 728	0.189

Note. M = adjusted mean, TS = teacher support, OGE = opposition to gifted education, SGS = support for gifted students, NGS = nurturance for gifted students, DT = differentiated teaching, LSRS = learning support for regular students.

## Data Availability

The data presented in this study are only available on request from the corresponding author.

## Appendix A

**Table A1:** Teacher well-being scale.

Items	
1.	Have confidence in one’s opinions
2.	Not afraid to voice opinions
3.	Decisions not influenced by other people
4.	Judge self by what is self-important
5.	No difficulty in arranging life
6.	Can manage the living situation
7.	Able to build a home and a lifestyle
8.	Good at managing responsibilities
9.	Have improved much as a person
10.	Have developed a lot as a person
11.	Learn, change, and grow in life
12.	Consider new experiences important
13.	Seen as loving and affectionate
14.	Described as a giving person
15.	Trust friends and being trusted
16.	Enjoy conversations with friends
17.	Have direction and purpose in life
18.	Not wander aimlessly through life
19.	Know what it is to accomplish
20.	Enjoy making plans for the future
21.	Confident and positive self-views
22.	Happy with how things have turned out
23.	Like most aspects of one’s personality
24.	Feel good about self
25.	In most ways my life is close to my ideal.
26.	The conditions of my life are excellent.
27.	I am satisfied with my life.
28.	So far I have gotten the important things I want in life.
29.	If I could live my life over, I would change almost nothing.

## Appendix B

**Table A2:** Teacher characteristics and competencies scale.

Items	
1.	Is highly intelligent
2.	Has cultural and intellectual interests
3.	Strives for excellence, high achievement
4.	Is enthusiastic about talent
5.	Relates well to talented people
6.	Has broad general knowledge
7.	Is mature, experienced, self-confident
8.	Can see things from students’ point of view.
9.	Is well organized, systematic, orderly
10.	Is imaginative, flexible, open to change, stimulating
11.	Is innovative and experimental, rather than conforming
12.	Recognizes individual differences
13.	Respects individuality, personal self-images, and personal integrity
14.	Can create a warm, safe, democratic environment
15.	Guides rather than coerces
16.	Has knowledge of the nature and needs of the gifted
17.	Can develop (or select) methods and materials for use with the gifted
18.	Is skilled in teaching higher thinking abilities, including creativity and problem solving
19.	Is adept at questioning techniques
20.	Is skilled in facilitating independent research
21.	Can direct individualized learning and teaching
22.	Is skilled in counseling gifted and talented youth
23.	Is skilled in group processes, teaching groups
24.	Can lead young people to successful accomplishments
25.	Can focus on process as well as product

Note. This scale was created by David Chan and Lai-kwan Chan. David Chan and Lai-kwan Chan are Program Founders and Honorary Program Director in the Program for the Gifted and Talented in Faculty of Education, The Chinese University of Hong Kong, respectively. They are academics who have been working in gifted education for many years (>30 years). In Project GIFT, David Chan was an Honorary Advisor and Lai-kwan Chan was a Co-Chief Principal Investigator of the project. She was the Project Operation Leader and Budget Holder from December 2016 to December 2018.
